# Description of *Clavibacter zhangzhiyongii* sp. nov., a phytopathogenic actinobacterium isolated from barley seeds, causing leaf brown spot and decline

**DOI:** 10.1099/ijsem.0.004786

**Published:** 2021-05-13

**Authors:** Qian Tian, Jiacheng Chuan, Xianchao Sun, Aiguo Zhou, Li Wang, Jixing Zou, Wenjun Zhao, Xiang Li

**Affiliations:** ^1^​ Institute of Plant Quarantine Research, Chinese Academy of Inspection and Quarantine, Beijing 100176, PR China; ^2^​ Canadian Food Inspection Agency (CFIA), Charlottetown Laboratory, Charlottetown, PE C1A 5T1, Canada; ^3^​ College of Plant Protection, Southwest University, Chongqing 400715, PR China; ^4^​ College of Marine Sciences, South China Agricultural University, Guangzhou 510642, PR China

**Keywords:** barley leaf brown spot and decline, barley seeds, *Clavibacter*, *Clavibacter zhangzhiyongii *sp. nov

## Abstract

*
Clavibacter michiganensis* is a Gram-stain-positive bacterium with eight subspecies, five of which have been redefined as different species on the basis of their genome sequence data. On the basis of the results of phylogenetic analysis of *dnaA* gene sequences, strains of members of the genus *Clavibacter* isolated from barley have been grouped in a separate clade from other species and subspecies of the genus *Clavibacter *. In this study, the biochemical, physiological, fatty acids and genetic characteristics of strains DM1^T^ and DM3, which represented the barley isolates, were examined. On the basis of results from multi-locus sequence typing and other biochemical and physiological features, including colony colour, carbon source utilisation and enzyme activities, DM1^T^ and DM3 are categorically differentiated from the aforementioned eight species and subspecies of the genus *Clavibacter *. Moreover, the results of genomic analysis reveal that the DNA G+C contents of DM1^T^ and DM3 are 73.7 and 73.5 %, respectively, and the average nucleotide identity (ANI) values between DM1^T^ and DM3 and other species and subspecies range from 90.4 to 92.0 %. The ANI value between DM1^T^ and DM3 is 98.0 %. These results indicate that DM1^T^ and DM3 are distinct from other known species and subspecies of the genus *Clavibacter *. Therefore, we propose a novel species, *C. zhangzhiyongii*, with DM1^T^ (=CFCC 16553 ^T^=LMG 31970^T^) as the type strain.

*Clavibacter* is an important bacterial genus that contains numerous plant pathogens of agricultural significance. According to conventional phenotypic and phylogenetic classification, *Clavibacter michiganensis *, for a substantial period of time, was the sole species of the genus *Clavibacter* and had five subspecies based on host specificity and morphogenetic features: *Clavibacter michiganensis* subsp. *michiganensis* causes tomato canker [1], *C. michiganensis* subsp. *sepedonicus* induces potato ring rot [2], *C. michiganensis* subsp. *insidiosus* causes wilting and stunting in alfalfa [3], *C. michiganensis* subsp. *nebraskensis* is responsible for Goss’s bacterial wilt and leaf blight in corn [4] and *C. michiganensis* subsp. *tessellarius* causes bacterial mosaic in wheat [2]. Recent technological advances have enabled the isolation and identification of novel subspecies of *C. michiganensis *, such as *C. michiganensis* subsp. *phaseoli *, which causes bacterial leaf yellowing in beans [5], and *C. michiganensis* subsp. *capsici *, which causes bacterial canker in pepper [6]. Some non-pathogenic subspecies closely related to *C. michiganensis* have been identified from tomato seeds produced in California and Chile, namely *C. michiganensis* subsp. *californiensis* and *C. michiganensis* subsp. *chilensis *, respectively [7]. The major plant pathogens among subspecies of *C. michiganensis* were recently reclassified as *Clavibacter sepedonicus *, *Clavibacter insidiosus *, *Clavibacter capsici *, *Clavibacter nebraskensis* and *Clavibacter tessellarius* on the basis of genomic differences [average nucleotide identity (ANI) and digital DNA:DNA hybridisation values] and multi-locus phylogenetic analysis [8]. The new classification system was supported by more evidence provided in a broader frame [9].

In 2017, an orange-pigmented, Gram-stain-positive coryneform bacterium was repeatedly isolated from barley seeds imported from Australia to China with DM1^T^ and DM3 as the representatives of these almost identical isolates. Isolates DM1^T^ and DM3, with similar sequences, were subsequently identified as representing members of the genus *Clavibacter* via 16S rRNA gene sequence comparisons. To determine the taxonomic position of these isolates from barley and their relationship to the existing species of the genus *Clavibacter *, 27 strains of members of the genus *Clavibacter *, including the type strains of eight species or subspecies with validly published names, were analysed and compared. Phylogenetic analyses were performed based on the *dnaA* gene. For multi-locus sequence typing analysis, the sequences of six housekeeping genes (*atpD*, *dnaK*, *gyrB*, *ppK*, *recA* and *rpoB*) of all the collected strains were amplified and sequenced. PCR amplifications were performed using primer pairs designed to amplify *dnaA* [10], *atpD*, *dnaK*, *gyrB*, *ppK*, *recA* [11] and *rpoB* [7] gene sequences (Table S1, available in the online version of this article), and the products were sequenced for phylogenetic analysis. The previously published genome sequences of several species of the genus *Clavibacter *, subspecies of *Clavibacter michiganensis* and the related *Rathayibacter iranicus* CFBP 807 and NCPPB 2253 strains were retrieved from the GenBank database (TableS2).

PCR amplification was performed in 50 µl reaction mixtures containing 25 µl of 2×PCR Master Mix (Biomed), 19 µl ddH_2_O, 2 µl of each primer (10 µM), and 2 µl DNA template with desired positive and negative controls. The primers and amplification conditions for each region are presented in Table S1. The PCR products were examined using 1.5 % agarose gel electrophoresis. Purification and bidirectional sequencing were performed by Sangon Biotech (Shanghai, PR China).

Sequences were assembled and edited using DNAMAN 7.0 (Lynnon). Multiple sequence alignments were performed using the ClustalW tool from mega X, and NJ trees were reconstructed using mega X, based on the Kimura two-parameter model. The percentage of replicate trees in which the associated taxa clustered together in the bootstrap test (1000 replicates) are shown next to the branches. *Rathayibacter iranicus* NCCPB 2253^T^ (accession number CP028130.1) was used as the outgroup for NJ trees based on partial *dnaA* sequences, whereas the six housekeeping gene sequences of *Rathayibacter iranicus* CFBP807 was used as outgroup for NJ trees (accession numbers: *atpD*, JX889817.1; *dnaK*, JX889995.1; *gyrB*, JX890084.1; *ppK*, JX890173.1; *recA*, JX890262.1; *rpoB*, JX889906.1). To render the results more intuitive, branches of the same species or subspecies were compressed using the compress subtree tool provided by mega X. The results of both *dnaA*-based phylogenetic analysis (Fig. 1) and multi-locus sequence typing analysis (Fig. 2) indicated that the isolates from barley can be grouped as a separate clade from other species of the genus *Clavibacter* and subspecies of *C. michiganensis *, indicating that barley-associated isolates DM1^T^ and DM3 differ from any of the known species and subspecies with validly published names. The complete phylogenetic trees are shown in Figs S1 and S2.

**Fig. 1.**
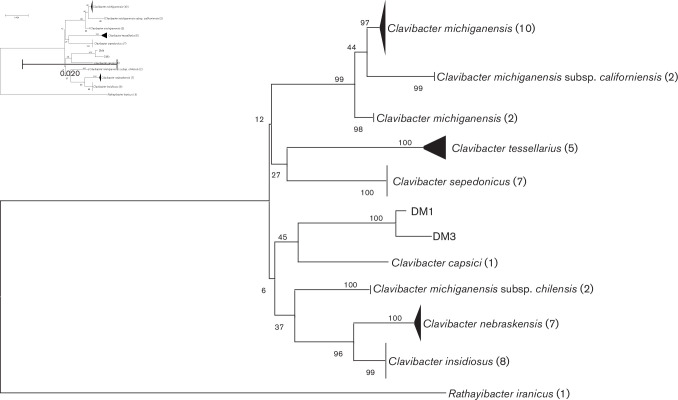
Phylogenetic analysis of *dnaA* sequences. NJ trees were based on partial *dnaA* sequences of 44 strains of members of the genus *Clavibacter* and rooted using *Rathayibacter iranicus* (accession number CP028130.1) as the outgroup. Bootstrap values (>50 %) are shown at branch points. Numbers following taxon names indicate numbers of isolates. The unit used for the scale bar is the evolutionary distance of the number of base substitutions per site.

**Fig. 2.**
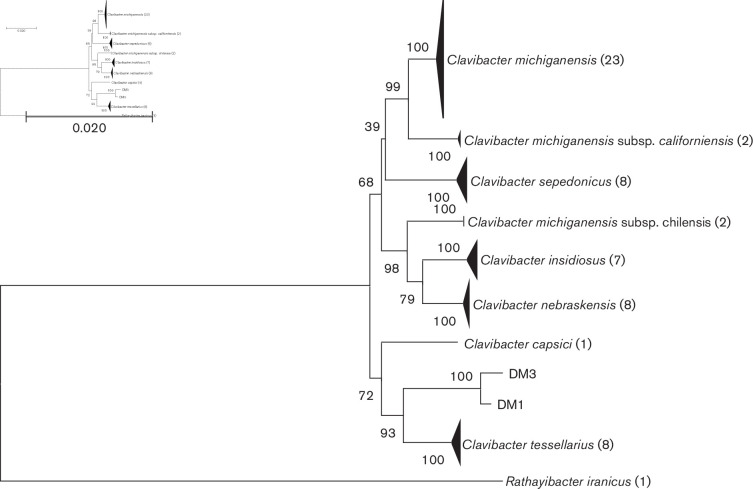
Multi-locus sequence analysis of concatenated *atpD*, *dnaK*, *gyrB*, *ppK*, *recA* and *rpoB* gene sequences. NJ trees were based on sequences of 60 strains of members of the genus *Clavibacter* and rooted using *Rathayibacter iranicus* CFBP 807 as the outgroup. Bootstrap values (>50 %) are shown at branch points. Numbers following taxon names indicate numbers of isolates. The unit used for the scale bar is the evolutionary distance of the number of base substitutions per site.

The GenBank accession numbers for the gene sequences of analysed are listed in Tables S2 (for the *atpD*, *dnaK*, *gyrB*, *ppk*, *recA* and *rpoB* sequences) and S3 (for the *dnaA* sequences).

To determine the distinct biochemical and physiological characteristics of DM1^T^ and DM3, various biochemical and physiological assays were conducted in comparison with other species and subspecies of the genus *Clavibacter* [5–7, 12]. DM1^T^ and DM3, with identical characteristics, were found to be Gram-stain-positive, coryneform, and non-motile. Colonies grown at 26 °C on nutrient agar (Difco, BD) were orange, round, and entire. Four types of medium were used to test the growth of the strains, namely CNS medium [13], CMM1 medium [14], Medium-6 (recommended by the Belgian Co-Ordinated Collections of Micro-Organisms/Laboratory of Microbiology) and TTC medium [15]. Bacterial growth on the different media was assessed via plate streaking and incubation at 26 °C for 3–7 days. Colony colour and morphology were observed. The results indicated that both DM1^T^ and DM3 could grow normally on all tested media.

For levan production assays, DM1^T^ and DM3 were incubated at 26 °C for 5 days on nutrient agar (Difco, BD), with 5 % sucrose [16]. For methyl red reaction assays, a bacterial suspension (10^8^ c.f.u. ml^−1^) was added to the media. After 5 days, 3–4 drops of methyl red were added to the culture [16]. The results for DM1^T^ and DM3 were all positive. NaCl tolerance tests were conducted by incubating bacteria at 26 °C in King’s B liquid medium with 1–8 % NaCl [16], and DM1^T^ grew in up to 5 % NaCl. To determine the maximum growth temperature, a bacterial suspension (10^8^ c.f.u. ml^−1^) was spread on King’s B agar medium and incubated at 26–37 °C for 7 days. The results indicated that DM1^T^ and DM3 could grow on this medium at 26–35 °C.

API Coryne and ZYM test strips were used to investigate carbohydrate fermentation and enzymic activities, respectively. The GEN III MicroPlate (Biolog) was used according to the manufacturer’s instructions to determine carbon source utilisation and chemical sensitivity. The results of all methods were compared with those from previous studies [5–7, 12]. Biochemical features differentiating other species and subspecies of the genus *Clavibacter* from the novel strain are shown in Table 1. Detailed results of API Coryne, API ZYM test strips and Biolog are shown in Tables S4–S6.

**Table 1.** Characteristics differentiating species of the genus *Clavibacter* and subspecies of *C. michiganensis* from strain DM1^T^ Taxa: 1, strain DM1^T^; 2, *C. michiganensis *; 3, *C. nebraskensis *; 4, *C. tessellarius *; 5, *C. insidiosus *; 6, *C. sepedonicus *; 7, *C. capsici *; 8, *C. phaseoli*; 9, *C. michiganensis* subsp. *californiensis *; 10, *C. michiganensis* subsp. chilensis*.* O, Orange; W, white; Y, yellow; +, >50 % positive results; w, 10–50 % positive results; −, <10 % positive results; v, variable; nd, no data available.

Characteristic	1	2*	3†	4†	5†	6†	7*	8‡	9§	10§
**Yellow or orange pigment**	O	Y	O/Y	O	W/Y	W	O	Y	Y/O	Y
**Colony type**	Domed, mucoid	Fluidal	Domed, mucoid	Domed, mucoid	Fluidal	Fluidal	Mucoid	Mucoid or fluidal	Mucoid	Mucoid
**Growth on**										
CNS	＋	＋	＋	＋	−	−	＋	nd	＋	＋
CMM1	＋	＋	＋	＋	＋	−	＋	nd	＋	＋
TTC	＋	＋	−	＋	＋	−	＋	＋	＋	＋
**Methyl red**	＋	v	v	−	＋	−	v	−	−	−
**Levan production**	＋	v	＋	＋	v	−	＋	−	＋	＋
**NaCl tolerance (%**)	5	5–6	5–7	nd	3–4	3	5–6	2–4	3–4	3–4
**Maximum growth temperature**	35	34–35	34	nd	31–32	30–32	33–34	34–35	35–36	35–36
**Nitrate reduction**	＋	−	−	−	−	−	＋	nd	−	−
**Hydrolysis of**										
Gelatine	＋	−	−	−	−	−	−	nd	−	−
**Enzyme activity:**										
Alkaline phosphatase	＋	w	＋	＋	−	v	＋	−	＋	＋
Trypsin	−	＋	−	−	−	−	＋	nd	−	−
α-Chymotrypsin	＋	＋	−	nd	−	−	＋	nd	−	−
Naphthol-AS-BI-phosphohydrolase	＋	w	−	−	−	−	−	nd	−	−
α-Mannosidase	−	−	−	−	＋	−	w	−	−	−
Cystine arylamidase	−	w	−	−	＋	v	w	nd	−	−
Pyrazinamidase	＋	−	−	−	−	−	−	nd	−	−
Urease	＋	−	−	−	−	−	−	nd	−	−

*Some of the data are from Oh *et al*. [6].

†Some of the data are Palomo *et al*. [12].

‡Some of the data are from González *et al*. [5].

§Some of the data are from Yasuhara-Bell *et al*. [7].

**Table 2.** Fatty acid compositions (percentages) of *Clavibacter capsici* PF008^T^, *C. michiganensis* subsp. *michiganensis* LMG 7333^T^ and DM1^T^

Strain		Anteiso			Iso	Norma		Others
	C_15 : 0_	C_15 : 1_	C_17 : 0_	C_15 : 0_	C_16 : 0_	C_17 : 0_	C_16 : 0_	
PF008^＊^	44.85	4.65	24.17	1.01	14.06	0.53	5.28	5.78
LMG7333^＊^	46.68	5.59	28.45	0.79	13.54	0.54	3.36	0.15
DM1	48.6	0.62	32.6	0.66	13.63	0.45	2.37	1.74

*Data from Oh *et al*. [6].

The fatty acid composition of DM1^T^ was analysed using gas chromatography. The major fatty acids were anteiso-C_15 : 0_ (12-methyl-tetradecanoic acid), anteiso-C_17 : 0_ (14-methyl-hexadecanoicacid), and iso-C_16 : 0_ (14-methyl-pentadecanoic acid; Table 2). These results were consistent with features of other members of the genus *Clavibacter* [17]. The entire genome sequence of DM1^T^ and the draft genome sequence of DM3 were obtained and annotated, and the DNA G+C contents of DM1^T^ and DM3 are 73.65 and 73.47 %, which are consistent with the characteristics of other members of the genus *Clavibacter* (Table S7) [17].

Average nucleotide identity (ANI) has emerged as a powerful genome-based criterion for establishing species identity amongst genetically related micro-organisms [8]. In this study, FastANI version 1.2 was used to measure the ANI values between DM1^T^, DM3 and 37 other strains of members of the genus *Clavibacter* from GenBank [18] (Fig. 3), and the ANI values among species of the genus *Clavibacter* were generally below the 96 % cutoff value (Fig. 3) for species delineation suggested by Richter and Rosselló-Móra [19]. The ANI value between DM1^T^ and DM3 is 98.0 % indicating that they represent the same species, and the ANI values of DM1^T^ and DM3 with all other members of the genus *Clavibacter* ranged from 90.4 to 92.0 % (Fig. 3), which are below the 96 % cut-off value for species delineation, indicating that DM1^T^ and DM3 represent the same novel species of the genus *Clavibacter *.

**Fig. 3.**
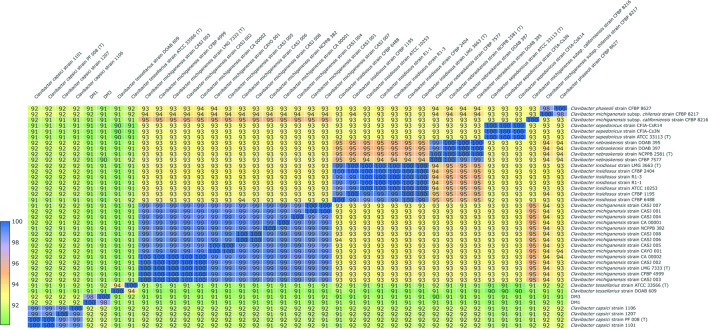
Average nucleotide identity heatmap of classified *Clavibacter* assemblies in NCBI and DM1^T^ and DM3 strain.

In term of pathogenicity, both DM1^T^ and DM3 were inoculated by soaking healthy barley seeds (cv Xiyin No.2) in bacterial suspensions (approximately 10^8^ c.f.u. ml^−1^) for 2 h and air-dried for two days. The seeds were sown, and the seedlings were observed over the course of a month. Inoculated plants were placed in an incubation chamber at 25 °C and 80 % relative humidity with the conditions of 19 °C (8 h) in dark and 23 °C (16 h) in light. Seeds soaked in distilled sterile water served as controls. Symptoms were observed two weeks after sowing. Initial symptoms of inoculated plants were leaf fading and yellowing, which sometimes co-occurred with brown spots. After four weeks, the tips of the leaves dried out. After five weeks, the entire leaf had dried out and withered (Fig. 4), whereas controls remained healthy. Colonies similar to DM1^T^ and DM3 were re-isolated from, and identified on, the symptomatic leaves of the inoculated plants.

**Fig. 4.**
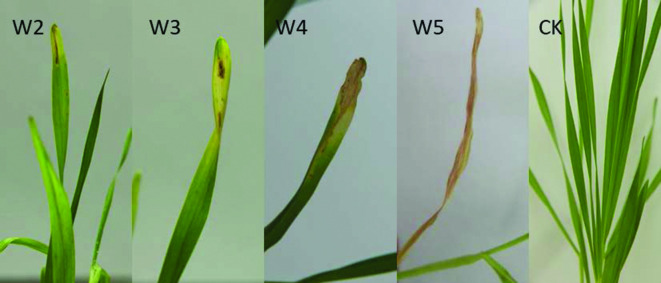
Disease symptoms in barley plants inoculated with DM1^T^. W2–W5, 2–5 weeks; CK, healthy control.

In conclusion, the biochemical and physiological characteristics data indicated that DM1^T^ and DM3 are members of the genus *Clavibacter *. Genome sequence comparison in the form of ANI values, the results of multi-locus phylogenetic analysis, and several biochemical traits indicate that these two strains differ from other species and subspecies of the genus *Clavibacter* with validly published names. Given their phylogenetic positions as well as their genotypic and chemotaxonomic features, strains DM1^T^ and DM3 can be classified as representing a novel species of the genus *Clavibacter *, for which the name *Clavibacter zhangzhiyongii* sp. nov. is proposed, with DM1 ^T^ (=CFCC 16553^T^=LMG 31970^T^) as the type strain.

## Description of *Clavibacter zhangzhiyongii* sp. nov.

*Clavibacter zhangzhiyongii* [zhang.zhi.yong′i.i. N.L. gen. n. *zhangzhiyongii,* named in honour of Professor Zhiyong Zhang (1927–1991), a well-known bacteriologist who dedicated his life to phytobacteriology and plant quarantine research in China].

Cells are Gram-stain-positive, non-spore forming, coryneform, non-motile aerobic bacteria without flagella. Expresses catalase activity, liquefies gelatine, produces levan, reduces nitrate, methyl red positive and grows on TTC, CMM1, and CNS media, produces orange colonies on common laboratory growth media. Colonies are round and entire, with diameters of 1–1.5 mm after 5 days of incubation on NA medium at 26 °C. Growth on King’s B agar medium occurs at temperatures of up to 35 °C and a maximum NaCl concentration of 5 %. The cells can use dextrin, maltose, trehalose, cellobiose, gentiobiose, sucrose, turanose, stachyose, raffinose, lactose, d-melebiose, β-methyl-d-glucoside, d-salicin, α-d-glucose, d-mannose, d-fructose, d-galactose, d-mannitol, *myo*-inositol, glycerol, l-aspartic acid, l-glutamic acid, pectin, l-malic acid, and acetoacetic acid. These bacteria show chemical sensitivity to 8 % NaCl, fusidic acid, d-serine, troleandomycin, rifamycin SV, minocycline, lincomycin, guanidine HCl, niaproof 4, vancomycin, tetrazolium violet, tetrazolium blue and sodium butyrate. They exhibit alkaline phosphatase, esterase (C4), esterase lipase (C8), urease, pyrazinamidase, leucine arylamidase, α-chymotrypsin, acid phosphatase, naphthol-AS-BI-phosphohydrolase, α‐galactosidase, β‐galactosidase, α‐glucosidase, and β‐glucosidase activities.

The type strain, DM1^T^ (=CFCC 16553^T^=LMG 31970^T^), was originally isolated from barley seeds imported from Australia into PR China and caused bacterial brown spot on and withering of barley leaves.

## Supplementary Data

Supplementary material 1
