# Supplementary material for: Description of Clavibacter zhangzhiyongii sp. nov., a phytopathogenic actinobacterium isolated from barley seeds, causing leaf brown spot and decline
Source: Int J Syst Evol Microbiol. 2021 May 13;71(5):004786. doi: 10.1099/ijsem.0.004786 (PMC8289203; doi:10.1099/ijsem.0.004786)
Supplement: Supplementary material 1 [file ijsem-71-4786-s001.pdf]

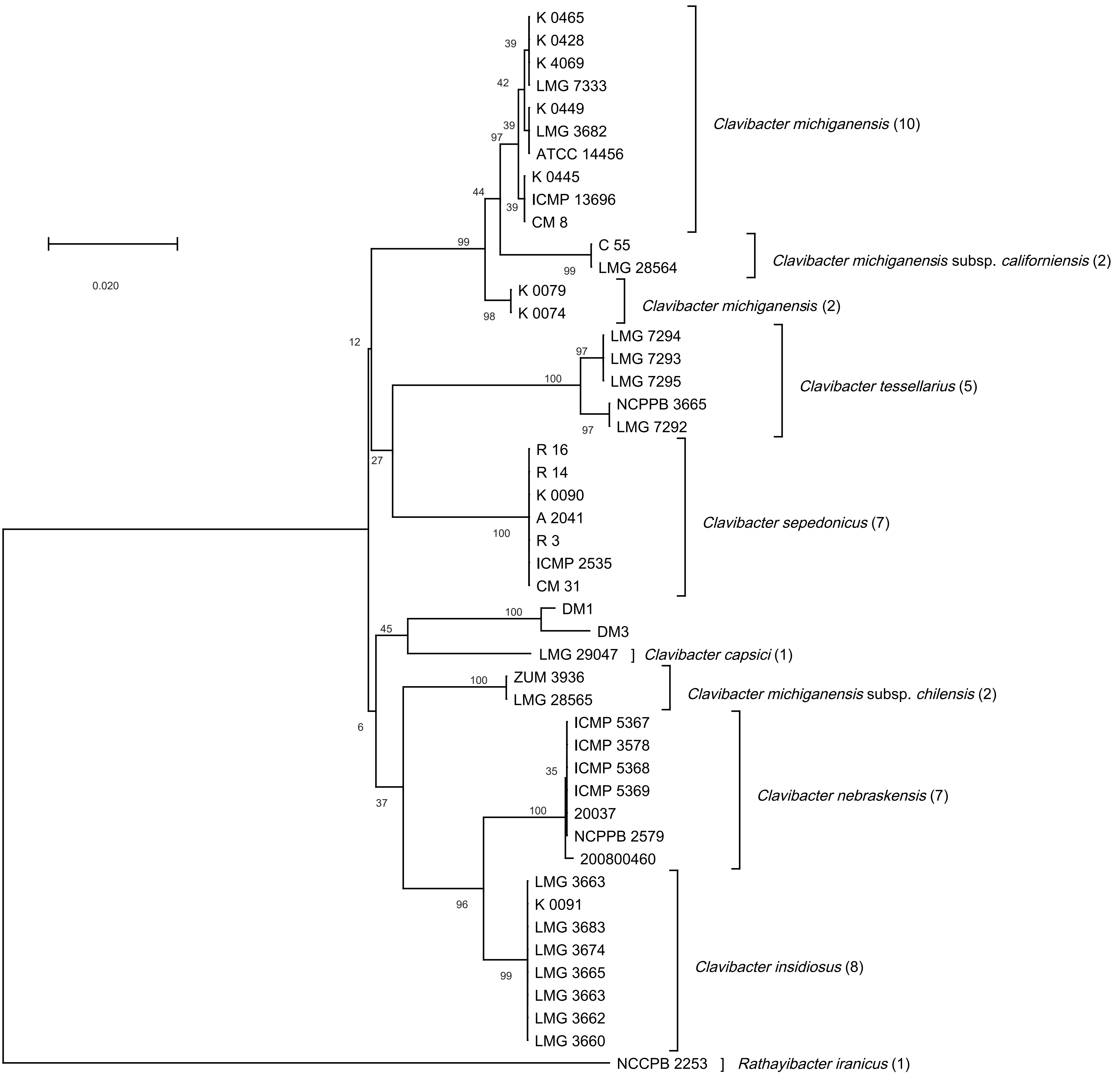

Fig. S1. Phylogenetic analysis of dnaA sequences (full).

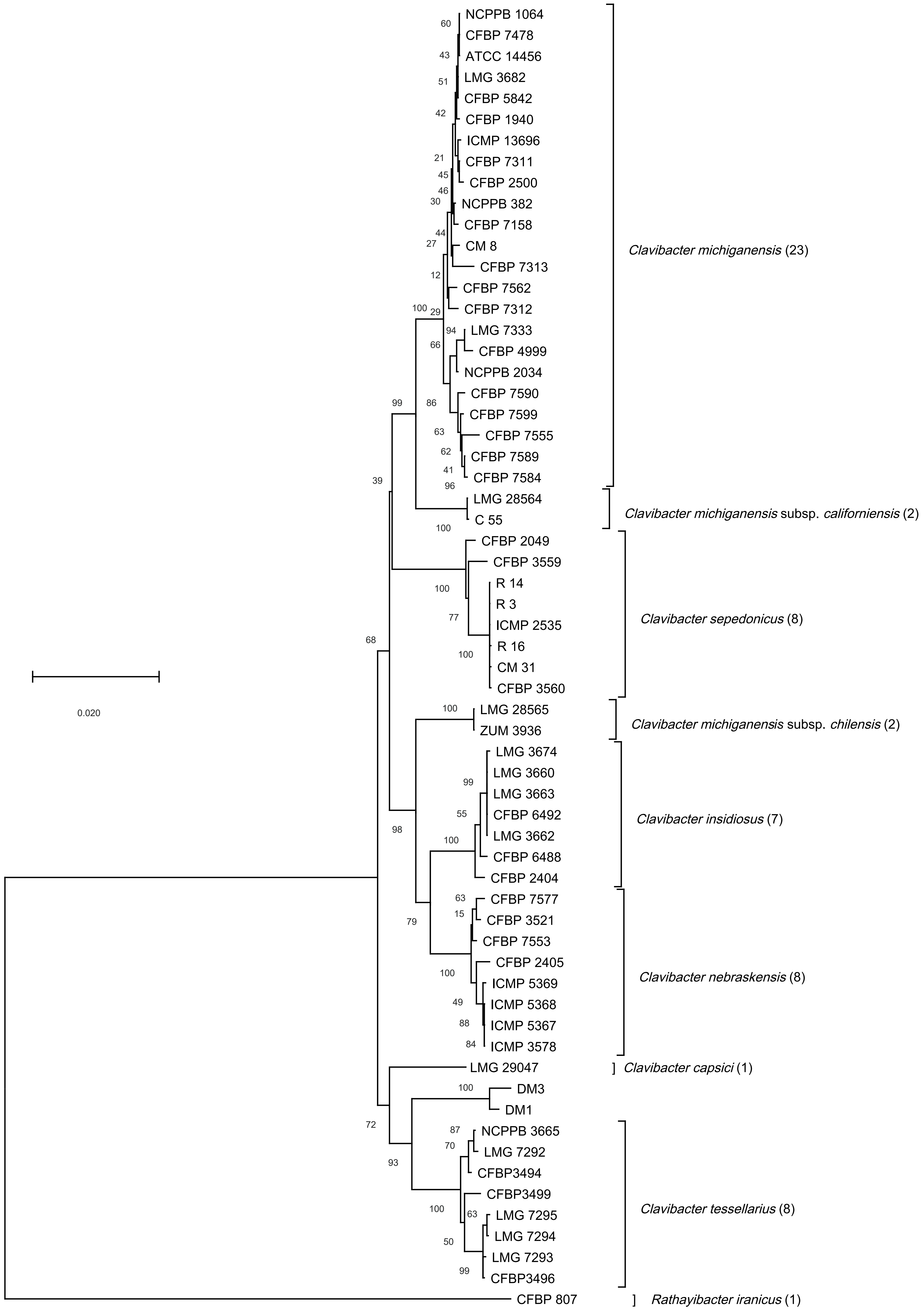

Fig. S2. Multi-locus sequence analysis of concatenated atpD, dnaK, gyrB, ppK, recA, and rpoB gene sequences (full).

Table S1. PCR primers used in this study.

Gene	Primers	Amplification conditions	Size (bp)	Source/reference
dnaA	dnaAF/dnaAR	94℃ 5 min; 94℃ 30 s, 61℃ 1 min, 72℃ 30 s, 35 cycles; 72℃ 10 min	933	Schneider *et al*. (2011)
atpD	atpdF/atpdR	94℃ 5 min; 94℃ 30 s, 60℃ 30 s, 72℃ 1 min, 35 cycles; 72℃ 10 min	697	
dnak	dnakF/dnakR	94℃ 5 min; 94℃ 30 s, 59℃ 30 s, 72℃ 1 min, 35 cycles; 72℃ 10 min	704	
gyrB	gyrbF/gyrbR	94℃ 5 min; 94℃ 30 s, 60℃ 30 s, 72℃ 1 min, 35 cycles; 72℃ 10 min	909	Jacques *et al*. (2012)
ppK	ppkF/ppkR	94℃ 5 min; 94℃ 30 s, 60℃ 30 s, 72℃ 1 min, 35 cycles; 72℃ 10 min	604	
recA	recaF/recaR	94℃ 5 min; 94℃ 30 s, 60℃ 30 s, 72℃ 1 min, 35 cycles; 72℃ 10 min	724	
rpoB	rpobF/rpobR	94℃ 5 min; 94℃ 30 s, 60℃ 30 s, 72℃ 1 min, 35 cycles; 72℃ 10 min	662	Yasuhara-Bell and Alvarez (2015)

Table S2. GenBank accession numbers for gene sequences used in MLST analysis.

Organism	Strain	GenBank accession numbers					
		atpD	*dnaK*	*gyrB*	*ppk*	*recA*	*rpoB*
C. michiganensis	ICMP 13696*	MT682281	MT661664	MT661692	MT682227	MT682256	MT661636
C. michiganensis	LMG 3682*	MT682282	MT661660	MT661681	MT682221	MT682250	MT661630
C. michiganensis	LMG 7333*	MT661703	MT661663	MT661679	MT682216	MT682245	MT661625
C. michiganensis	CM 8*	MT661705	MT661662	MT661678	MT682234	MT682263	MT661643
C. michiganensis	ATCC 14456*	MT661704	MT661661	MT661680	MT682235	MT682264	MT661644
C. michiganensis	NCPPB 2034†	JX889819	JX889997	JX890086	JX890175	JX890264	JX889908
C. michiganensis	NCPPB 1064†	JX889818	JX889996	JX890085	JX890174	JX890263	JX889907
C. michiganensis	NCPPB 382†	JX889820	JX889998	JX890087	JX890176	JX890265	JX889909
C. michiganensis	CFBP 7599†	JX889815	JX889993	JX890082	JX890171	JX890260	JX889904
C. michiganensis	CFBP 7590†	JX889812	JX889990	JX890079	JX890168	JX890257	JX889901
C. michiganensis	CFBP 7589†	JX889811	JX889989	JX890078	JX890167	JX890256	JX889900
C. michiganensis	CFBP 7584†	JX889809	JX889987	JX890076	JX890165	JX890254	JX889898
C. michiganensis	CFBP 7562†	JX889799	JX889977	JX890066	JX890155	JX890244	JX889888
C. michiganensis	CFBP 7555†	JX889797	JX889975	JX890064	JX890153	JX890242	JX889886
C. michiganensis	CFBP 7478†	JX889784	JX889962	JX890051	JX890140	JX890229	JX889873
C. michiganensis	CFBP 7313†	JX889773	JX889951	JX890040	JX890129	JX890218	JX889862
C. michiganensis	CFBP 7312†	JX889772	JX889950	JX890039	JX890128	JX890217	JX889861
C. michiganensis	CFBP 7311†	JX889771	JX889949	JX890038	JX890127	JX890216	JX889860
C. michiganensis	CFBP 7158†	JX889768	JX889946	JX890035	JX890124	JX890213	JX889857
C. michiganensis	CFBP 5842†	JX889763	JX889941	JX890030	JX890119	JX890208	JX889852
C. michiganensis	CFBP 4999†	JX889761	JX889939	JX890028	JX890117	JX890206	JX889850
C. michiganensis	CFBP 2500†	JX889753	JX889931	JX890020	JX890109	JX890198	JX889842
C. michiganensis	CFBP 1940†	JX889740	JX889918	JX890007	JX890096	JX890185	JX889829
C. sepedonicus	R 3*	MT682268	MT661647	MT661676	MT682209	MT682243	MT661618
C. sepedonicus	R 14*	MT682267	MT661646	MT661675	MT682208	MT682242	MT661617
C. sepedonicus	R 16*	MT682266	MT661645	MT661674	MT682207	MT682241	MT661616
C. sepedonicus	CM 31*	MT682270	MT661648	MT661698	MT682233	MT682262	MT661642
C. sepedonicus	ICMP 2535*	MT682269	MT661649	MT661697	MT682232	MT682261	MT661641
C. sepedonicus	CFBP 3560†	JX889760	JX889938	JX890027	JX890116	JX890205	JX889849
C. sepedonicus	CFBP 3559†	JX889759	JX889937	JX890026	JX890115	JX890204	JX889848
C. sepedonicus	CFBP 2049†	JX889741	JX889919	JX890008	JX890097	JX890186	JX889830
C. tessellarius	NCPPB 3665*	MT682276	MT661650	MT661682	MT682210	MT682236	MT661619
C. tessellarius	LMG 7295*	MT682277	MT661656	MT661683	MT682217	MT682246	MT661626
C. tessellarius	LMG 7294*	MT682278	MT661657	MT661684	MT682218	MT682247	MT661627
C. tessellarius	LMG 7293*	MT682279	MT661658	MT661685	MT682219	MT682248	MT661628
C. tessellarius	LMG 7292*	MT682280	MT661659	MT661686	MT682220	MT682249	MT661629
C. tessellarius	CFBP 3499†	JX889757	JX889935	JX890024	JX890113	JX890202	JX889846
C. tessellarius	CFBP 3496†	JX889756	JX889934	JX890023	JX890112	JX890201	JX889845
C. tessellarius	CFBP 3494†	JX889755	JX889933	JX890022	JX890111	JX890200	JX889844

C. nebraskensis	ICMP 5369*	MT682288	MT661670	MT661693	MT682228	MT682257	MT661637
C. nebraskensis	ICMP 5368*	MT682289	MT661671	MT661694	MT682229	MT682258	MT661638
C. nebraskensis	ICMP 5367*	MT682290	MT661672	MT661695	MT682230	MT682259	MT661639
C. nebraskensis	ICMP 3578*	MT682291	MT661673	MT661696	MT682231	MT682260	MT661640
C. nebraskensis	CFBP 7577†	JX889807	JX889985	JX890074	JX890163	JX890252	JX889896
C. nebraskensis	CFBP 7553†	JX889796	JX889974	JX890063	JX890152	JX890241	JX889885
C. nebraskensis	CFBP 3521†	JX889758	JX889936	JX890025	JX890114	JX890203	JX889847
C. nebraskensis	CFBP 2405	JX889744	JX889922	JX890011	JX890100	JX890189	JX889833
C. insidiosus	LMG 3674*	MT682283	MT661665	MT661687	MT682222	MT682251	MT661631
C. insidiosus	LMG 3663*	MT682285	MT661667	MT661689	MT682224	MT682253	MT661633
C. insidiosus	LMG 3662*	MT682286	MT661668	MT661690	MT682225	MT682254	MT661634
C. insidiosus	LMG 3660*	MT682287	MT661669	MT661691	MT682226	MT682255	MT661635
C. insidiosus	CFBP 6488†	JX889765	JX889943	JX890032	JX890121	JX890210	JX889854
C. insidiosus	CFBP 2404†	JX889743	JX889921	JX890010	JX890099	JX890188	JX889832
C. insidiosus	CFBP 6492†	JX889766	JX889944	JX890033	JX890122	JX890211	JX889855
C. michiganensis subsp. *chilensis*	ZUM 3936†	KF663891	KF663925	KF663949	KF663973	KF663997	KF664021
C. michiganensis subsp. *chilensis*	LMG 28565*	MT682272	MT661652	MT661677	MT682212	MT682238	MT661621
C. michiganensis subsp. *californiensis*	C 55†	KF663873	KF663907	KF663931	KF663955	KF663979	KF664003
C. michiganensis subsp. *californiensis*	LMG 28564*	MT682273	MT661653	MT661702	MT682213	MT682239	MT661622
C. capsici	LMG 29047*	MT682271	MT661651	MT661700	MT682211	MT682237	MT661620
Clavibacter sp.	DM1*	MT682265	MT682204	MT682205	MT682206	MT682201	MT682202
Clavibacter sp.	DM3*	MW701417	MW701419	MW701420	MW701421	MW701422	MW701423
Rathayibacter iranicus	CFBP 807†	JX889817	JX889995	JX890084	JX890173	JX890262	JX889906

* Gene sequences obtained in this study.

† The previously published gene sequences.

Table S3. GenBank accession numbers for *dnaA* gene sequences used in this study.

No.	Bacterial species	Strains	GenBank accession ID
1	*C. michiganensis*	K 0448[†]	HM181213.1
2	*C. michiganensis*	K 0469[†]	HM181230.1
3	*C. michiganensis*	K 0465[†]	HM181227.1
4	*C. michiganensis*	K 0449[†]	HM181216.1
5	*C. michiganensis*	K 0428[†]	HM181201.1
6	*C. michiganensis*	K 0079[†]	HM181170.1
7	*C. michiganensis*	K 0074[†]	HM181168.1
8	*C. michiganensis*	LMG 7333[*]	MT505329
9	*C. michiganensis*	LMG 3682[*]	MT505327
10	*C. michiganensis*	CM 8[*]	MT505330
11	*C. michiganensis*	ATCC 14456[*]	MT083923
12	*C. michiganensis*	ICMP 13696[*]	MT505328
13	*C. sepedonicus*	R 16[*]	MT661706
14	*C. sepedonicus*	R 14[*]	MT661707
15	*C. sepedonicus*	K 0090[†]	HM181287.1
16	*C. sepedonicus*	A 2041[†]	KF663900
17	*C. sepedonicus*	R 3[*]	MT661708
18	*C. sepedonicus*	ICMP 2535[*]	MT498631
19	*C. sepedonicus*	CM 31[*]	MT661712
20	*C. nebraskensis*	NCPPB 2579[†]	KF663899
21	*C. nebraskensis*	200800460[†]	KF663898
22	*C. nebraskensis*	20037[†]	KF663897
23	*C. nebraskensis*	ICMP 5369[*]	MT498627
24	*C. nebraskensis*	ICMP 5368[*]	MT498628
25	*C. nebraskensis*	ICMP 5367[*]	MT498629
26	*C. nebraskensis*	ICMP 3578[*]	MT661710
27	*C. insidiosus*	LMG 3683[*]	MT498630
28	*C. insidiosus*	LMG 3674[*]	MT472807
29	*C. insidiosus*	LMG 3665[*]	MT472808
30	*C. insidiosus*	LMG 3663[*]	MT661711
31	*C. insidiosus*	LMG 3662[*]	MT472809
32	*C. insidiosus*	LMG 3660[*]	MT472810
33	*C. insidiosus*	K 0091[†]	HM469685.1
34	*C. tessellarius*	LMG 7295[*]	MT498636
35	*C. tessellarius*	LMG 7294[†]	KF663901
36	*C. tessellarius*	LMG 7293[*]	MT498638
37	*C. tessellarius*	LMG 7292[*]	MT498635
38	*C. tessellarius*	NCPPB 3665[*]	MT661709

39	*C. michiganense* subsp. *californiensis*	C 55†	KC416011
40	*C. michiganense* subsp. *californiensis*	LMG 28564*	MT498634
41	*C. michiganense* subsp. *chilensis*	ZUM 3936†	KC416020
42	*C. michiganense* subsp. *chilensis*	LMG 28565*	MT498633
43	*C. capsici*	LMG 29047*	MT498632
44	*Clavibacter* sp.	DM1*	MT682203
45	*Clavibacter* sp.	DM3*	n.d.
46	*Rathayibacter iranicus*	NCCPB 2253†	CP028130.1

* Gene sequences obtained in this study.

† The previously published gene sequences.

Table S4. Enzymatic activities according to the API® ZYM test strip.

Test	1*	2*	3※	4※	5※	6※	7$	8¶	9¶	10
Alkaline phosphatase	+	+	-	+	v	+	-	w	+	+
Esterase (C 4)	w	w	+	+	+	+	ND	+	+	+
Esterase lipase (C 8)	w	w	+	+	+	+	ND	+	+	+
Lipase (C 14)	-	-	-	-	-	-	ND	-	-	-
Leucine arylamidase	+	+	+	+	+	+	ND	+	+	+
Valine arylamidase	-	-	-	-	v	-	ND	-	-	-
Cystine arylamidase	-	-	+	-	v	-	+	w	w	-
Trypsin	-	-	-	-	-	-	ND	+	+	-
α-chemotrypsin	-	-	-	-	-	ND	ND	+	+	+
Acid phosphatase	+	+	+	+	v	+	ND	+	+	+
Naphthol-AS-BI-phosphohydrolase	w	-	-	-	-	-	ND	w	-	+
α-galactosidase	+	+	+	v	-	+	+	+	+	+
β-galactosidase	+	+	+	v	+	+	ND	+	+	+
β-glucuronidase	-	-	-	-	-	-	ND	-	-	-
α-glucosidase	+	+	+	v	+	+	ND	+	+	+
β-glucosidase	+	+	+	-	+	+	-	+	+	+
N-acetyl-β-glucosaminidase	-	-	-	-	-	-	ND	-	-	-
α-mannosidase	-	-	+	-	-	-	-	-	w	-
α-fucosidase	-	-	-	-	-	-	ND	-	-	-

1, *C. michiganensis* subsp. *californiensis*; 2, *C. michiganensis* subsp. *chilensis*; 3, *Clavibacter insidiosus*; 4, *Clavibacter nebraskensis*; 5, *Clavibacter sepedonicus*; 6, *Clavibacter tessellarius*; 7, *Clavibacter phaseoli*; 8, *C. michiganensis*; 9, *Clavibacter capsici*; 10, DM1.

+, >50% positive results; w, 10-50% positive results; -, <10% positive results; v, variable; ND, not determined.

*1~2 Date for Jarred Yasuhara-Bell1 and Anne M. Alvarez (2015).

※ 3~6 Data from Palomo *et al.* (2006).

$7 Date for Ana J. Gonza´lez and Estefanı´a Trapiello (2014).

¶8~9 Date for Eom-Ji Oh *et al.* (2016).

Table S5. Enzymatic activities and fermentation of carbohydrates according to the API® Coryne test strip.

Test	1*	2*	3*	4*	5*	6*	7*	8$	9
**Nitrate reduction**	-	-	-	-	-	-	-	+	+
**Enzymatic activity:**									
Pyrazinamidase	-	-	-	-	-	-	-	-	+
Pyrrolidonyl arylamidase	-	-	-	-	-	-	-	-	-
Alkaline phosphatase	+	+	w	+	+	w	+	+	+
β-glucuronidase	-	-	-	-	-	-	-	-	-
β-galactosidase	+	+	w	+	+	w	+	+	+
α-glucosidase	+	+	+	+	+	w	+	+	+
N-acetyl-β-glucosaminidase	-	-	-	-	-	-	-	-	-
β-glucosidase	+	+	w	+	+	w	+	+	+
Urease	-	-	-	-	-	-	-		+
Catalase	+	+	+	+	+	+	+	+	+
**Hydrolysis of:**									
Gelatin	-	-	-	-	-	-	-	-	+
**Fermentation of:**									
D-glucose	-	-	-	-	-	-	-	-	-
D-ribose	-	-	-	-	-	-	-	-	-
D-xylose	-	-	-	-	-	-	-	-	-
D-mannitol	-	-	-	-	-	-	-	-	-
D-maltose	-	-	-	-	-	-	-	+	-
D-lactose	-	-	-	-	-	-	-	-	-
D-saccharose (sucrose)	-	-	-	-	-	-	-	-	-
Glycogen	-	-	-	-	-	-	-	-	-

1, *C. michiganensis* subsp. *californiensis*; 2, *C. michiganensis* subsp. *chilensis*; 3, *Clavibacter insidiosus*; 4, *C. michiganensis*; 5, *Clavibacter nebraskensis*; 6, *Clavibacter sepedonicus*; 7, *Clavibacter tessellarius* ； 8, *Clavibacter capsici*; 9, DM1.

+, >50% positive results; w, 10-50% positive results; -, <10% positive results.

*1~7 Date for Jarred Yasuhara-Bell1 and Anne M. Alvarez (2015).

$8 Date for Eom-Ji Oh et al. (2016).

Table S6. Carbon source utilization and chemical sensitivity according to the BIOLOG test system.

Test	1*	2*	3*	4*	5*	6*	7*	8	9	10
**Carbon source utilization:**										
Dextrin	+	+	+	+	+	-	+	+	+	w
D-maltose	+	+	w/v	+	+	-	+	+	w	+
D-trehalose	+	+	-	+	+	-	+	+	w	w
D-cellobiose	+	+	+	+	+	+	+	+	+	+
Gentiobiose	+	+	-	+	+	-	+	+	+	+
Sucrose	+	+	+	+	+	-	+	+	+	+
D-turanose	+	+	+	+	+	-	+	+	+	+
Stachyose	+	+	-	+	+	-	+	+	w	w
D-raffinose	+	w	-	+	-	+	w/-	-	-	-
α-D-lactose	+	+	+	+	+	-	+	+	+	+
D-melibiose	+	+	-	+	+	-	+	w	w/-	-
β-methyl-D-glucoside	+	+	-	+	+	-	+	+	+	+
D-salicin	+	+	-	+	+	-	+	+	+	+
N-acetyl-D-glucosamine	-	+	-	-	-	-	-	w	+	-
N-acetyl-β-D-mannosamine	-	w/-	-	-	-	-	-	-	w/-	-
N-acetyl-D-galactosamine	-	-	-	-	-	-	-	-	-	-
N-acetyl-neuraminic acid	-	-	-	-	-	-	-	-	-	-
α-D-glucose	+	+	+	+	+	+	+	+	+	+
D-mannose	+	+	+	+	+	+	+	+	+	+
D-fructose	+	+	+	+	+	+	+	+	+	+
D-galactose	+	+	+	+	+	-	+	+	+	+
3-methyl glucose	-	-	+/v	-	-	-	-	-	-	-
D-fucose	w	-	w/v	w/v	-	-	-	w	-	-
L-fucose	-	-	+/v	w	-	-	-	-	-	-
L-rhamnose	-	+	w/v	-	-	-	-	w	-	-
Inosine	w/v	+	-	+/w	-	-	+	w	w/-	-
D-sorbitol	+/w	+	w	+	+	-	+	w	w/-	w/-
D-mannitol	+	+	+	+	+	+	+	+	+	+
D-arabitol	-	-	-	-	-	-	-	-	-	-
Myo-inositol	+	+	+	+	+	-	+	+	w/-	-
Glycerol	+	+	+	+	+	-	+	+	+	+
D-glucose-6-phosphate	-	-	-	w/-	-	-	-	w	-	w/-
D-fructose-6-phosphate	-	-	-	w	-	-	-	w	w/-	w/-
D-aspartic acid	-	-	-	-	-	-	-	w	-	-
D-serine	-	-	-	-	-	-	-	-	-	-
Gelatin	-	-	-	-	-	-	w/v	-	-	-
Glycyl-L-proline	-	-	-	-	-	-	-	w	-	-
L-alanine	v	+	-	w/v	+	-	+	+	-	w

L-arginine	-	-	-	-	-	-	-	w	-	-
L-aspartic acid	+	w	+	+	w	-	-	w	-	w/-
L-glutamic acid	+	+	-	+	w	-	+/w	w	-	w/-
L-histidine	-	-	-	-	-	-	-	-	-	-
L-pyroglutamic acid	-	-	-	-	-	-	-	w	-	-
L-serine	-	+	-	+	+	-	+	+	w/-	+
Pectin	+	+	+	+	+	+	+	+	w	w
D-galacturonic acid	-	-	-	-	-	-	-	-	w/-	w/-
L-galactonic acid lactone	-	-	-	-	-	-	-	-	-	-
D-gluconic acid	+	+	-	+	+	+	+	+	+	+
D-glucuronic acid	-	-	-	-	-	-	-	-	-	-
Glucuronamide	w	-	+/w	+/w	-	-	-	w	-	w/-
Mucic acid	-	-	-	w	-	-	-	w	-	-
Quinic acid	+	-	+	+	-	-	-	w	-	-
D-saccharic acid	-	-	-	-	-	-	-	w	-	-
p-hydroxy-phenylacetic acid	-	-	-	-	-	-	-	-	-	-
Methyl pyruvate	-	w	-	-	-	-	-	-	-	w
D-lactic acid methyl ester	-	-	-	w/-	-	-	-	-	-	-
L-lactic acid	-	w	-	-	-	-	-	-	-	-
Citric acid	-	+	-	+	w	-	+	+	w	w
α-ketoglutaric acid	-	w	-	w	-	-	-	w	-	w/-
D-malic acid	-	-	-	w	-	-	-	w	-	w/-
L-malic acid	+	+	+	+	-	-	+	+	w	+
Bromo-succinic acid	-	+	-	+	-	-	+	-	w/-	w
Tween 40	-	+	+	+	+	-	+	w	w/-	w
γ-amino-butyric acid	-	-	-	-	-	-	-	-	-	w
α-hydroxy-butyric acid	-	-	-	-	-	-	+	w	-	-
β-hydroxy-D,L-butyric acid	-	-	-	-	-	-	+	-	-	w/-
α-keto-butyric acid	-	w	-	-	-	-	w/v	-	-	w
Acetoacetic acid	+	+	+	+	+	+	+	+	+	+
Propionic acid	-	-	-	w	w/-	-	w/-	w	-	w
Acetic acid	w	-	+	+	w/-	-	+/w	+	w	+
Formic acid	-	-	-	-	-	-	-	w	-	-
**Chemical sensitivity:**										
pH6	+	+	+	+	+	+	+	+	+	+
pH5	+/w	+	-	+	w	+	+	w	-	-
1% NaCl	+	+	+	+	+	w	+	+	+	+
4% NaCl	+/w	+	-	+	v	-	+	w	w/-	w/-
8% NaCl	-	+/w	-	-	-	-	+/v	w	-	-
1% Sodium lactate	+	+	-	+/v	+	-	+	+	+	+
Fusidic acid	-	-	-	-	-	-	-	-	-	-
D-serine	-	-	-	-	-	-	-	-	-	-

Troleandomycin	-	-	-	-	-	-	-	-	-	-
Rifamycin SV	-	-	-	-	-	-	-	-	-	w
Minocycline	-	-	-	-	-	-	-	-	-	-
Lincomycin	-	-	-	-	-	-	-	-	-	w/-
Guanidine HCl	-	-	-	+	-	-	-	w	-	w/-
Niaproof 4	-	-	-	-	-	-	-	-	-	-
Vancomycin	-	-	-	-	-	-	-	-	-	-
Tetrazolium violet	-	-	-	-	-	w	-	-	+	+
Tetrazolium blue	-	-	-	-	-	-	-	-	-	w
Nalidixic acid	+	+	+	+	+	+	+	+	+	w
Lithium chloride	+	+	w	+	w	-	+	+	+	+
Potassium tellurite	+	+	+	+	+	+	+	-	+	+
Aztreonam	+	+	+	+	+	+	+	+	+	+
Sodium butyrate	-	+	w	-	-	-	+	w	-	w/-
Sodium bromate	w	+	-	+	+	-	+	-	w	w

1, *C. michiganensis* subsp. *californiensis*; 2, *C. michiganensis* subsp. *chilensis*; 3, *Clavibacter insidiosus*; 4, *C. michiganensis*; 5, *Clavibacter nebraskensis*; 6, *Clavibacter sepedonicus*; 7, *Clavibacter tessellarius*; 8, *Clavibacter capsici*; 9, DM1; 10, DM3.

+, >50% positive results; w, 10-50% positive results; -, <10% positive results; v, variable.

*1~7 Data retrieved from Jarred Yasuhara-Bell and Alvarez (2015).

Table S7. Comparison of genomes of different Clavibacter species.

Characteristic	DM1	DM3	*Cm*. subsp. *californiensis*[*]	*Cm*. subsp. *chilensis*[*]	*Cm*. strain LMG 26808[£]	*C. michiganensis*[$]	*C. nebraskensis*[‡]	*C. sepedonicus*[¥]	*C. capsici*[†]	*C. insidiosus*[††]	*C. phaseoli*[‡‡]	*C. tessellarius*[**]
Chromosome size (Mb)	3.1	3.0	3.2	3.0	3.5	3.3	3.1	3.3	3.1	3.4	3.1	3.3
GC content (%)	73.7	73.5	72.6	73.6	72.0	72.7	73.0	72.6	73.2	72.7	73.5	73.7
Plasmids[¥]	nd	nd	nd	nd	pC1 (⩽70 kb)	pCM1 (27 kb)<br>pCM2 (70 kb)	-	pCS1 (50 kb)<br>pCSL1 (95 kb)	pCM1 (40kb)<br>pCM2 (146kb)	-	-	-

Symbols: -, none; nd, not determined.

[¥]Plasmid content varies depending on the strain.

[£]Data obtained from Zaluga et al. (2014).

[$]Data obtained from Gartemann et al.(2008).

[‡]Data obtained from Eichenlaub et al.(2010).

[¥]Data obtained from Bentley et al. (2008).

[*]Data obtained from Yasuhara-Bell et al. (2014).

[†]Data obtained from Oh et al. (2016).

[††]Data obtained from Li et al. (2017).

[‡‡]Data obtained from Osdaghi et al. (2018).

[**]Data obtained from Yuan et al. (2017).
